# Ethical climate as moderator in the indirect relationship between dark triad traits and organizational pride among polish employees

**DOI:** 10.1038/s41598-026-51762-x

**Published:** 2026-05-05

**Authors:** Marcin Wnuk

**Affiliations:** Department of Work and Organizational Psychology ,Faculty of Psychology and Cognitive Sciences, Adam Mickiewicz University in Poznań, ul. Szamarzewskiego 89/AB, 60-568 Poznań, Poland

**Keywords:** Dark triad, Organizational pride, Ethical climate, Need for power, Person–organization fit, Psychology, Human behaviour

## Abstract

Studies have considered the roles of antecedents of organizational pride. However, they have not yet investigated the personality and motivational factors and potential interactions between the ethical environment, personality traits, and employees’ needs in explaining organizational pride. This study fills the gap within the person-organization fit approach by examining the relationship between dark triad personality traits and organizational pride, mediated by the opportunities to satisfy the need for power and moderated by perceptions of an instrumental ethical climate. The cross-sectional research included 1,000 participants (mean age = 41.06; 54.9% men and 45.1% women) employed on a full-time, permanent contract in companies of various sizes in Poland. A positive relationship between narcissism and organizational pride was found (B = 0.30, *p* = 001), as was a negative relationship between Machiavellianism and organizational pride (B = − 0.26, *p* = 003), and between psychopathy and organizational pride (B = − 0.29, *p* = 001). Perception of the instrumental climate moderated the relationship between narcissism and the opportunities to satisfy the need for power (t = 3.19; *p* = 009), Machiavellianism and the opportunities to satisfy the need for power (t = 2.93.; *p* = 003), psychopathy and the opportunities to satisfy the need for power (t = 2.94; *p* = 033), as well as opportunities to satisfy the need for power and organizational pride (t = 5.57; *p* = 000). Indirect positive relationships between narcissism and Machiavellianism and organizational pride, mediated by opportunities to satisfy the need for power and amplified by a perception of an instrumental ethical climate, were confirmed. The beneficial function of an instrumental ethical climate and opportunities to satisfy the need for power of narcissistic and Machiavellian employees’ organizational pride were discussed. Practical implications were formulated regarding the recruitment and selection of work candidates and their development in terms of dark triad features and their congruency with instrumental ethical climate and possibilities to satisfy their need for power.

## Introduction

Organizational pride is a prosocial and desirable attitude in the workplace leading to positive outcomes in the workplace, such as organizational commitment^1,2^, job satisfaction^3^, task performance, organizational citizenship behaviors^4^, customer-oriented behaviors^5^, employees’ voluntary pro-environmental behaviors, organizational embeddedness^6^, and lower turnover intentions^7^. Recent studies have explored organizational antecedents of organizational pride, including distributive justice, work conditions, organizational identification, management support^1^, organizational trust, visionary leadership^8^, perception of corporate social responsibility^9,10^, organizational competence, and virtuousness^3^.

However, personality traits that can potentially facilitate or impede the shaping of organizational pride and the impact of workplace context in expressing the behaviors and filling the needs resulting from these traits have never been tested. This research target was employees who manifested the dark triad personality traits. Recently, only one study considered the mutual connections between possessing the dark triad traits and the social work environment (competitive psychological climate at work) from the perspective of potentially matching each other, but without considering the effect of this fit for the organization^11^.

Three ways of the reciprocal relationship between personality traits and social environment in the workplace are possible: evocation, manipulation, and selection^12^. In accordance with the evocation effect^12^, a previous study showed the influence of employees with dark triad traits unconsciously to elicit predictable reactions in their social environments^11^. Another way to achieve this aim is manipulation^12^, which is an intentional and preferable strategy used in social interactions among the dark triad trait workers. Finally, the selection process relies on the choice of the work environment, which characterizes the attributes similar to those possessed by employees to maximize match within the reciprocal connection. For example, Spurk and Hirschi^11^ found this effect in employees possessing narcissistic and psychopathic traits who consistently perceived a competitive psychological climate even after the job change.

In my study, through the analysis of their motivational structure and ethical climate consistent with their preferences, two potential factors salient to organizational pride were proposed: opportunities to satisfy the need for power and instrumental ethical climate, which reflects values and requirements consistent with employees’ expectations who characterize by the dark triad traits. Due to the individual-oriented values^13^, the instrumental ethical climate is suited and desirable to employees with dark triad traits, whose common and crucial characteristics are selfishness^14^, exploitation of others^15–18^, rivalry^19^, and pursuit to attain power as the leading value they pursuit^20–25^. The theoretical ground for this research was the person-organization fit (PO-Fit) theory, which emphasizes the benefits of matching employees and organization, manifested in the favourable attitude toward the organization and commitment to it^26^. PO-Fit theory distinguishes two types of matching: complementary and supplementary. Within a complementary fit, the resources and characteristics of employees are essential in an organization that lacks them. In this case, employees offer the organization something valuable that they possess to fill a gap within the organization. Supplementary fit refers to the similarities between employees and the organization in terms of values, needs, business purposes, ethical norms, and standards, among others^26^.

This research is grounded in the supplementary fit of characteristics of employees with dark triad traits and the ethical norms of the organization as premises for creating opportunities to satisfy the need and build organizational pride. It was the first attempt to verify the propensity of employees with dark triad personality traits to feel organizational pride and whether conditions such as perception of instrumental workplace climate and opportunities to meet the need for power to achieve this aim. Following the results of Spurk and Hirschi’s^11^ findings, I assumed the expression of dark triad traits as a consequence of the perception of instrumental ethical climate will be positively related to opportunities to fill the need for power and, in turn, to organizational pride. Besides some commonalities, a competitive psychological climate differs from an instrumental climate because it makes employee rewards dependent on the results of performance, comparing them to those of other employees^27^. An instrumental ethical climate fosters ethical decisions that prioritize individual well-being over the interests of community^13^. Competitive psychological climate focuses on performance criteria as the main indicator of employees’ value, whereas instrumental ethical climate emphasizes ethical norms, rules, and values embedded in processes, procedures, and politics, which emphasize self-interest as the leading standard of ethical action. A common element of competitive psychological climate and instrumental ethical climate is a focus on individual needs and aspirations, in contrast to community goodness, which can strengthen rivalry between employees and may lead to harmful consequences^28–30^.

This study can contribute to the area of investigation and HRM practices, connecting the expressions of dark triad traits as an undesirable phenomenon in the workplace with organizational pride, highlighting the conditions that need to be met to attain this goal. Firstly, answering the question about the predispositions of employees with dark triad traits to express identification with the organization in the form of organizational pride is a significant topic for HRM. Secondly, finding the appropriate ethical climate they expect, which will enable them to satisfy their need for power, is an opportunity to build pride in the organization. In reference to recruitment process gives an opportunity to suit better this kind of candidate to an ethical workplace environment and even a job position to avoid the cost connected with the selection failure and time devoted to it. From the employment stability point of view, it increases the probability of staying in the organization and prevents potential replacement. Thirdly, from the promotion perspective, this can direct this process on the possibility of filling the needs that are crucial for this group of employees. Finally, showing the potential commonalities and differences between every dark triad trait in achieving organizational pride implies the same or differing ways of interventions targeted at improving this area.

## Dark triad and organizational pride: direct path

The dark triad is a construct that encompasses a set of subclinical personality traits such as narcissism, Machiavellianism, and psychopathy^31^ with the leading element of antisocial motivation. Within this conception, dark triad traits are interpreted as overlapping, but characterizing significant distinctiveness. Prominent features of narcissism are grandiosity, entitlement, and self-centeredness. Machiavellianism encompasses manipulation, cynicism, and strategic exploitation, and psychopathy is manifested by impulsivity, callousness, and lack of remorse^31^. Common characteristics of all dark triad traits are the desire for dominance and power^20–25^, aggressiveness^32^, a tendency to exploit others^15–18^, pathological selfishness^14^, manipulation and malevolency^15–17^, problems with empathy^14,33^, and lack of humility^14,34,35^. In recent decades, a debate has emerged regarding the core of the dark triad in relation to the HEXACO model of personality, and promising candidates for this role have been explored. At this stage of the debate, research outcomes have indicated that the low honesty-humility dimension of HEXACO is the best factor of personality that reflects the core of the dark triad conception^36,37^. For example, Vize et al.^34^ reported that Honesty-Humility correlated negatively with all three Dark Triad factors in the range between 0.83 and 0.99.

The impact of the dark triad traits on workplace attitudes is complex and determined by the considered trait, used construct measures, and research sample^25^. Additionally, contextual factors related to sociocultural differences have their reflections in expressions dark triad traits in the workplace^25^. For example, narcissistic workers in the USA are more prone to counterproductive work behavior, organizational citizenship behavior, and voice behavior, are more engaged and satisfied with their job, and present fewer indications of exhaustion, turnover intention, and boredom at work^32^. A lack of cultural differences was confirmed regarding the relationships between psychopathy and workplace attitudes, but the strongest cultural diversity was observed in the reference connections between Machiavellianism and the used indicators of attitudes in the workplace^32^.

Possessing psychopathic and Machiavellian inclinations leads to detrimental outcomes or is irrelevant to phenomena in the workplace, but a narcissistic attitude at work can be favorable for an organization. This regularity regards most but not every attitude, behavior, emotional state, and experience of employees with dark triad traits. For some workplace outcomes, like moral disengagement^38^, the dark triad’s detrimental impact is irrespective of the concrete trait, but for others, like job satisfaction, turnover intentions, and organizational commitment, narcissism can be favorable—in contrast to Machiavellianism and psychopathy. Ma et al. ^39^ reported that narcissistic employees were less motivated to turnover intentions compared to Machiavellian and psychopathic workers, who had more motivation to leave an organization. Additionally, narcissistic employees, compared to Machiavellian and psychopathic, were job-satisfied. Taking into account the cited research, it is valuable to investigate if employees with dark triad traits can express pride in the organization, and explore potential differences in this issue between narcissistic workers and Machiavellian and psychopathic workers.

Organizational pride encompasses pride in being a member of an organization as a collective attitudinal element resulting from the desire to belong to the organization and common membership as something meaningful and salient. It refers to positive identification with the organization and possessing desirable attributes, pursuing achievements, and possessing an awareness of contributions to its development and success^8^. A second element of organizational pride regards positive emotional states like pride, joy, and happiness yielded from organizational membership, which are assessed as valuable and evoke admiration. Organizational pride is an element desirable in employees as a vital motivational factor that can lead to a company’s success. Both components of organizational pride reflect a positive attitude, a bond with the organization, and positive emotions regarding the company.

In some studies, affective organizational commitment was queried as a consequence of organizational pride, and they found a moderate predictive role of pride in the organization in explaining affective commitment to the organization^1,2^. Taking into account the pivotal role of organizational pride in developing an emotional bond with the organization, it seems necessary to determine which motivational factors are connected with achieving this pride in employees with dark triad traits. Previously, dark triad traits have not been investigated in the context of organizational pride. The appropriate point of reference to consider this issue is recent results regarding the connection between employees’ dark triad traits and their organizational commitment. Both of these phenomena shared the identification with the company and the expression of positive feelings toward it. On the other hand, besides the potential overlap between organizational identification, affective commitment, and organizational pride as similar constructs, they differ from each. Organizational identification is based on an individual’s perception of their own self-concept as intertwined with the organization’s identity^40^. Affective commitment, reflecting emotional attachment, positive feelings, and involvement with the organization, and a strong desire to remain^41^. Organizational pride is a positive feeling of admiration, honor, or glory derived from the organization’s achievements, reputation, or perceived excellence.^8^ For example, organizational pride is not necessary for organizational identification, but can be a sufficient antecedent of affective commitment.

Lyons et al.^42^, in a sample of Amazon’s Mechanical Turk employees, found a positive connection between narcissism and affective organizational commitment and a negative connection between Machiavellian tactics and Machiavellian view with this variable. The role of psychopathy in organizational commitment was irrelevant. In turn, among employees in South Korea, Machiavellianism was negatively correlated with affective organizational commitment^43^. In Michel and Bowling’s^44^ research, narcissism positively predicted organizational commitment.

The motivational difference between narcissism in comparison to Machiavellianism and psychopathy in the prediction of organizational commitment in narcissistic employees is that narcissistic employees stem from the fact that narcissistic employees are more capable and more interested in building organizational affective commitments than individuals with psychopathic and Machiavellian traits^42,43^. Besides common elements of motivational structure, the individuals with narcissistic characteristics differ from individuals with psychopathic and Machiavellian traits regarding affiliation needs. In previous research, narcissism was found to be positively related to the need for affiliation, but Machiavellianism and psychopathy negatively connected with it^41,23,44^. Employees with narcissistic traits are prone to satisfy belonging needs^45^ as the primary need at work^46^ due to unstable self-esteem, insecure self-image^47^, and vulnerability to interpersonal rejection^48^. Additionally, being part of something significant and special, and contributing to this venture, can enhance pride and grandiose tendencies in employees with narcissistic characteristics, creating the possibility of being admired—a relevant element they pursue and desire^49^. Narcissism has two forms: grandiose and vulnerable. This first type of narcissism can be defined as manipulative, phallic, overt, egotistical, oblivious, exhibitionistic, and psychopathic. Vulnerable narcissism encompasses craving, contact-shunning, hypervigilant, and shy^50^. Both narcissism manifestations share a lack of agreeableness and unstable self-esteem^17,51^ inflated self-esteem in reference to individuals with grandiose narcissism and low self-esteem among vulnerable narcissism. The main differences between them regarding emotional stability, extraversion, impulsiveness, and susceptibility to stress, anxiety, and depression^17,51,52^. In this context, the grandiose narcissism is more adaptive and functional. It is possible that grandiose narcissism would be positively related to organizational pride in comparison to the vulnerable form of narcissism, but in this study, overall narcissism without differentiating these two types was employed. Narcissism was verified by the Dirty Dozen Scale, which appropriately reflects both grandiose and vulnerable narcissistic characteristics. On the other hand, the Dirty Dozen Scale has some limitations, especially low discriminant validity between dark triad traits^17,18^, and refers to exploitation of others as a common element relevant to every manifestations of dark triad^18,53^. Finally, some concerns have been raised in research regarding the multidimensional structure of dark triad traits^18,54^. Taking into consideration that concurrent tool to the Dirty Dozen Scale, such as Short Dark Triad (SD3), also has some limitations^18,54,55^, but contain almost three times more questions, I decided to use the Polish adaptation of the Dirty Dozen Scale.

Unwillingness to build bonds with the organization among workers with Machiavellian and psychopathic traits can be the effect of their cynicism,^31,]^, emotional coldness^31,56^, as well as their propensity to feel high negative and low positive affect^57^, which can impede the building of affective commitment. Cynicism as a character trait, especially characteristic of Machiavellian individuals^53^, differs from organizational cynicism as an attitude toward the organization^58,59^, but can strengthen that. Cynical employees tend to react to an organization cynically and express a cynical attitude toward it^60^. Organizational cynicism is a phenomenon opposite to organizational pride because it is connected with the presence of recognition and admiration toward the organization but with the negative image of it as calculated, unreliable, and two-faced, that deserves reluctance, indifference, and hostility^58,59^.

Based on the above conclusions, I hypothesized that narcissism is directly positively related to organizational pride, but Machiavellianism and psychopathy are directly negatively associated with organizational pride (Hypothesis 1).

### Dark triad and opportunities to satisfy the need for power: moderating roles of instrumental ethical climate

Some common elements in all dark triad manifestations drive workplace actions and make some goals attractive and desirable. Employees with dark triad traits share the desirability of power^61–63^, which has been reflected in many studies, creating a consistent picture of this topic. Positive relationships between dark triad traits and the driving by-power motive have been observed in studies regardless of the sample and the measures used to verify psychopathy, Machiavellianism, and narcissism^20–25^. For example, Semenyna and Honey^63^ confirmed the propensity of students with dark triad traits to use two styles of functioning as manifestations of dominance, namely ruthless self-advancement and dominant leadership. Also, in employees possessing dark triad traits, competitiveness can be a way to control and dominate others. Jonason et al.^64^ showed that workers with dark triad traits have a tendency to perceive the workplace as focused on competitiveness.

Prior studies have shown the relevancy of the power motive but have not indicated what kind of workplace conditions are favorable to satisfy this need^11^. Still, a topic vital to recognize remains the opportunities for employees with dark triad traits to express their tendency to dominate and fill their need for power in the concrete ethical environment at work, which can facilitate this process. Lewin emphasizes that behavior is a function of possessing some personality traits and the cultural environment one is in^65^. That means that workplace ethical climate can work as an activator and booster of some character traits or block and inhibit their expressions. Trait activation theory^66^ indicates that the probability of a given behavior occurring in the workplace depends on the organization, social, and/or task cues that come from the organizational environment as factors activating traits, whose expressions are desirable, expected, and perceived as appropriate. The stronger the incentive is, the bigger the chance of the presence of the attitude resulting from the trait activation caused by an action. Actions perceived by the employee as valued and expected by the organization have a larger chance of appearing if they are coherent with the employee’s traits, values, and needs. As Spurk and Hirschi^11^ showed, the selection strategy is dominant in narcissistic and psychopathic employees, who perceive a competitive work social environment in work independence of their place of employment. It corresponds with the fact of stability in the search for a similar social environment suited to personality traits as more predictable, manageable, and secure^67^. On the other side, they^11^ revealed that besides the change in perception of competitive psychological climate positively predicted by all triad traits, perception of this type of climate strengthened the dark triad traits, which is consistent with trait activation theory^66^.

One source of information regarding expected attitudes in the workplace came from the perception of ethical climate, which indicates an ethical standard of conduct in making ethical decisions^13^. Ethical standards are points of reference, especially in complex, ambiguous, and conflicted ethical situations. Victor and Cullen^13^ distinguish five types of ethical standards, which reflect five ethical climates in organizations: law and codes, independence, caring, instrumental, and rules. Within the law and codes ethical climate, the company establishes its ethical standards of conduct based on universal ethical codes, such as law or the Bible. In an ethical climate of independence, the point of reference for ethical decisions in the workplace is the set of rules and ethical values that are characteristic of individual employees. The caring and ethical climate characterizes the pursuit of the well-being of the greatest possible number of employees, with a strong emphasis on communal values and cooperation. The instrumental climate is antagonistic to a caring climate, focusing on the needs of individuals, valuing individual achievements, and encouraging rivalry. Finally, in the rules ethical climate, ethical standards are created and implemented within the organization, being autonomous, characteristic, and tailored to its needs^13^.

I decided to investigate the instrumental climate for two main reasons. First, it corresponds with rival drive,^19^ selfcentered orientation^14^, and the possibility of exploiting workmates^15–18^ as crucial for narcissistic, Machiavellian, and psychopathic individuals and induces the context of competition preferred by this group of employees^11^. Secondly, it implements favorable social environment conditions for filling the need for power^60–62^.

The leading motive that constitutes an instrumental ethical climate is commitment to the focus on caring for the well-being and needs of individuals, even at the expense of the rest of the employees.^13^ This individualistic approach encourages employees to concentrate on self-interest, a fight for power, control, and dominance, giving permission to exploit others, and the possibility to express confronting and aggressive behaviors. In the line of trait activation theory^66^, this shows that this type of workplace climate suits dark triad workers because they have a tendency to interpret the cues they receive as incentives to realize selfish goals^14^. Selfishness is an overriding value shared by instrumental ethical climate and individuals with dark triad traits behaviors.^14^ Previous research found evidence for egoistic purpose and desires as common and crucial in the motivational structure of individuals possessing the dark triad traits. For example, Dinić et al.^14^ confirmed that pathological selfishness shares 86% of the total common variance with dark triad traits^21^. Kaufman et al.^62^ found at least a moderate correlation between narcissism, Machiavellianism, and psychopathy and three types of selfishness: egocentric, pathological, and adaptive. Analogous outcomes were reported by Raine and Uh^68^ in two different research samples.

It is worth emphasizing that perception of instrumental climate has harmful effects in the workplace and may be related to unethical and antisocial behaviors, like unethical pro-organizational behavior^69^, lower moral awareness^70^, stealing, lying, pretending the sickness, giving the gifts/favors, falsify reports and exaggerate benefits^71^. Susceptibility to the counterproductive and deviant behavior of employees with dark triad traits^72^ provides additional arguments for the adequacy of instrumental climate to this group of workers’ aspirations and behavioral tendencies.

The presented evidence on the grounds of P-O Fit theory^26^, leads us to expect a stronger positive relationship between dark triad personality traits and filling the need for power in case of a stronger perception of instrumental climate.

I assumed that the perception of an instrumental ethical climate moderates the relationship between dark triad traits and the opportunities to satisfy the need for power (Hypothesis 2). I anticipated that the perception of an ethical climate would strengthen the relationship between the dark triad traits of personality and the opportunities to satisfy the need for power.

### Opportunities to satisfy the need for power and organizational pride - moderating role of instrumental ethical climate

The consequences in an organization of an employee’s need for power realization is a relatively rare topic of inquiry and most often appears in the context of employee empowerment as a benevolent phenomenon at work or the influence of power managers possess on employee need attitudes. Previous research has indicated worker’s empowerment as a positive antecedent of job satisfaction^73^, organizational commitment^74,75^, and job engagement^76^ and as a factor that may prevent burnout^73,77^, stress^78^, and turnover intention^79^.

Studies focused on employees’ need for power and dominance in the workplace have shown the different effects of realizing this need on turnover intention^80^, work/family conflict and job-induced tension^81^, job satisfaction^82^, physical symptoms^83^, citizenship and participating behaviors^84^, or abusive supervision^85^. For example, Zhang et al.^86^ found that in high-tech enterprises, employee power was positively related to organizational commitment directly and indirectly through out-of-role behaviors.

The effects of satisfaction of a need for power can partially depend on the main motive behind this need. Moon et al.^87^ showed that personalized power, as a desire for power for one’s own ends, is correlated with unethical decision-making, counterproductive workplace behaviors, and a lower level of the trait of honesty—humility. Power driven by prosocial motives to help others was irrelevant to unethical decision-making and counterproductive workplace behaviors. In the case of individuals with dark triad traits, the motive underly the actions directed to achieve power has a personal, non-social character and is based on their selfish desires^14^, and can be associated with positive results, especially since self-oriented behaviors are welcomed in an instrumental climate. The actions of these employees are connected to the desire for power and can be strengthened and awarded by the organization, even if their motivation is antisocial. Detection of the possibility of pursuit of power and the satisfaction of this need is identified with the organizational permission and cues interpreted as incentives to be involved in power achieve attitudes, because of the perception of instrumental climate.

Following the above arguments, I expected that the perception of an instrumental ethical climate moderates the relationship between the opportunities to satisfy the need for power and organizational pride (Hypothesis 3). I expected that the perception of an ethical climate would strengthen the relationship between opportunities to satisfy the need for power and organizational pride.

### Dark triad and organizational pride: indirect path through opportunities to satisfy the need for power moderated by instrumental ethical climate

Following the P-O Fit approach^26^, I assumed that the possibility of filling the need for power is a positive predictor of feeling pride in the organization because of the significance of this motive for employees with dark triad traits^60–62^, who may identify the ability to satisfy their need for power with the opportunities created by the organization and perceive the value of power as also substantial for the company. Employees might associate the possibility of realizing power, dominance, influence, and control with the organization’s will. This would not only give permission but also encourage power manifestations within an instrumental ethical climate, giving the potential premise to find pride in this approach. This relationship would be amplified by incentives coming from the perception of an instrumental ethical climate directed towards individualistic behaviors and caring about self-interest^13^. It will lead to a situation in which the organization’s needs and values, manifested within the instrumental ethical climate, seem congruent with the employee’s opportunities to satisfy the need for power, creating the conditions to do so more effectively. Opportunities to fill the need for power, in turn, will increase the employees’ organizational pride, and this effect will depend on how strong the perception of the instrumental climate is. The stronger perception of instrumental climate will amplify the positive relationship between satisfaction with the need for power and organizational pride.

Considering the previous findings, I hypothesized that the indirect relationship between dark triad traits and organizational pride is mediated by the opportunities to satisfy the need for power and moderated by the perception of an instrumental ethical climate (Hypothesis 4).

## Method

### Study design and participants

The study had a cross-sectional design. Four inclusion criteria were applied: being an adult, living and working in Poland, possessing a full-time, permanent contract of employment, and having been hired for at least three years in actual work. No financial incentives for participating were offered. The research received ethical approval from the Ethics Committee at the University of Silesia in Katowice (KEUS475/03.2024. All procedures performed in studies involving human participants were in accordance with the ethical standards of the institutional and/or national research committee and with the 1964 Helsinki declaration and its later amendments or comparable ethical standards. The participants were informed about the anonymity of the research and the possibility to withdraw from the study at any point without giving any reasons and without any consequences. Participants provided informed consent online before starting the survey.

### Setting

An external research company, Openfield, within the Manulo research panel was responsible for identifying respondents who met the inclusion criteria listed above. Openfield is a specific Polish market research agency that conducts broad consumer, scientific, and business studies. The study was conducted in August and September 2024. They collected data by sending invitations to participate in the research to individuals in their database who filled out the questionnaire on the online platform. Consistent with the research company’s status, participants received points for completing the questionnaire, and after accumulating enough points, they could exchange them for various rewards. Besides the inclusion criteria, the sample was representative, reflecting the distribution of socio-demographic variables in the Polish population.

### Conceptual model and variables

**Fig. 1:**
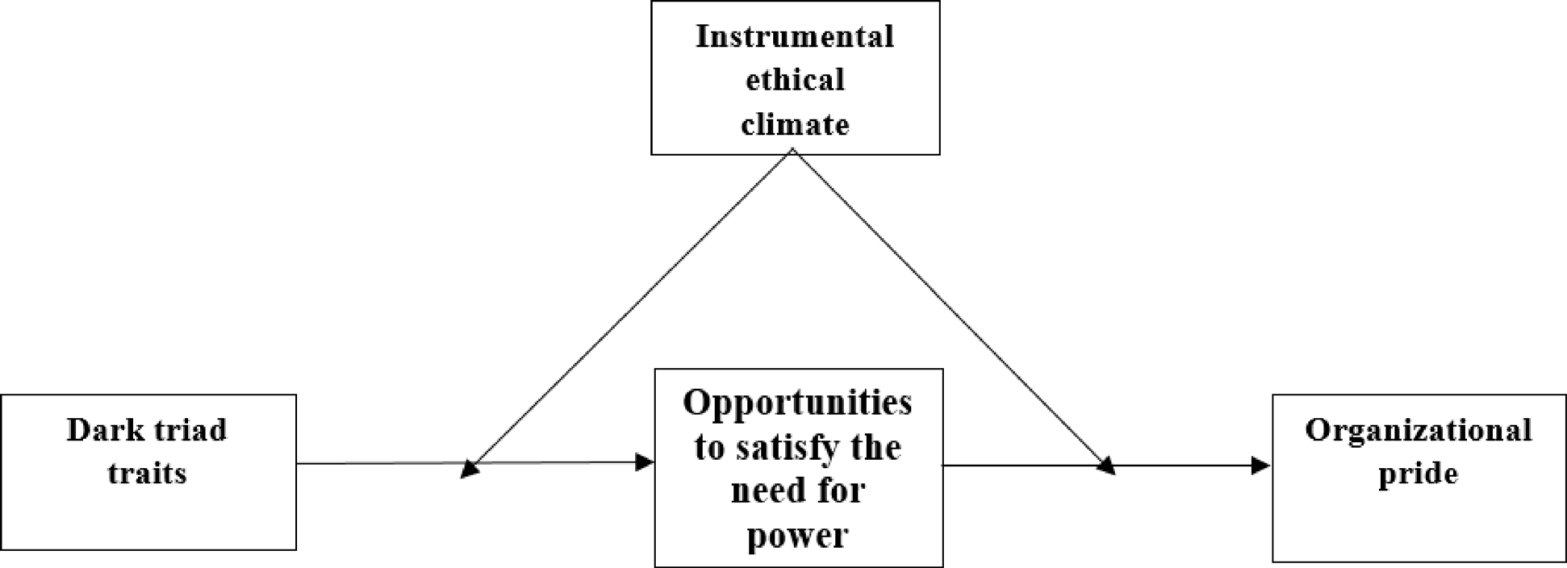
Theoretical model.

The conceptual model is presented in Fig. 1. The model examined moderated mediation with dark triad traits as independent variables, the opportunities to satisfy the need for power in the role of potential mediating variable, instrumental ethical climate and as moderator, and organizational pride as dependent variable. To the model, the following controlled variables were introduced: sex, age, education, seniority, seniority in present work, and company size. Also, the other two dark triad traits were simultaneously controlled in the regression equation for every tested dark triad trait as an independent variable. As a previous study suggests, dark triad traits are more characteristic of men and younger individuals compared to women and older individuals^88,89^. Also, in smaller companies, manifestations of dark triad traits seem to be less visible and transparent than in larger ones. In turn, instrumental ethical climate in Polish organizations is negatively related to age and educational level, and positively correlated with company size^30^. Finally, in recent research, organizational pride was not related to age, education, and sex^8^, but can be positively correlated with seniority in a present work^2^.

## Sample

In the study, 1000 employees from different companies in various industries located in Poland participated. Among the research participants, 663 were representatives of the public sector, 326 were employed in the private sector, and 11 were representatives of non-governmental organizations (see Table 1). The study sample was balanced in terms of sex and company size. The mean age of the participants was just over 40 years, with a mean seniority of almost 19 years. The vast majority had a high school education and higher education. Over half of the participants were married, and above 75% were employed in non-managerial positions.

**Table 1:** Sample characteristics (*N* = 1000).

Variable	*n* (%)
*Sex*	
Men	549 (54.9)
Women	451 (45.1)
Age (in years)	41.06 (10.76)^a^
*Marital status*	
Single	211 (21.1)
Married	550 (55)
Informal relationship	136 (13.6)
Informal relationship (living separately)	42 (4.2)
Widower	15 (1.5)
Divorced	53 (5.3)
Separated	1 (0.1)
Number of children	1.16 (1.10)^a^
Number of dependents	1.67 (1.72)^a^
*Education level*	
Primary	11 (1.1)
Lower secondary	5 (0.5)
Vocational	95 (9.5)
High school	432 (43.2)
Higher	447 (44.7)
Ph.D.	10 (1.0)
Seniority (in years)	18.77 (48.11)^a^
*Monthly income before taxes*	
1,000–1,999 zl	15 (1.5)
2,000–2,999 zl	37 (3.7)
3,000–3,999 zl	192 (19.2)
4,000–5,499 zl	285 (28.5)
5,500–6,999 zl	174 (17.4)
7,000–8,499 zl	89 (8.9)
8.500–9,999 zl	46 (4.6)
10.000-11.999 zł	28 (2.8)
12.000 zł and more	35 (3.5)
I prefer not to respond to this question.	99 (9.9)
Working hours per week	46.55 (24.28)^a^
*Form of employment*	
Full-time contract	1000 (100)
Seniority in present work (in years)	9.40 (859)^a^
*Sector of employment*	
Public	663 (66.3)
Private	326 (32.6)
Non-governmental organization	11 (1.1)
*Organization size*	
1–9 employees	135 (13.5)
10–49 employees	273 (27.3)
50–249 employees	258 (25.8)
250 employees and more	334 (33.4)
*Managerial position*	
No	755 (75.5)
Yes, a lower-level manager	109 (10.9)
Yes, a middle-level manager	93 (9.3)
Yes, a higher-level manager	31 (3.1)
Self-employment	12 (1.2)

^a^ For continuous variables means and standard deviations (in parenthesis) are presented.

### Measurement

#### Instrumental ethical climate

An instrumental work climate was verified by four items encompassing this dimension from the Polish version of the Ethical Work Climate Questionnaire^30^. This measure examines employee perceptions of an ethical work climate, distinguishing five types of ethical climate: instrumental, caring, law and code, rules, and independence^13^. In this study, only items regarding instrumental ethical climate were used (“In this company, people protect their own interests above”, “In this company, people are mostly out for themselves“). The items were rated on a five-point Likert scale (from 1 = strongly disagree to 5 = strongly agree). Satisfactory reliability of this measure, as well as divergent and convergent validity, were confirmed in samples of Polish employees^30^. In this study, the reliability of the instrumental ethical climate subscale, as calculated by Cronbach’s alpha, was 0.81.

### Opportunities to satisfy the need for power

Opportunities to satisfy the need for power was examined with the items being indicators of this dimension from the Opportunities to Satisfy Needs and Needs Satisfaction (OSNANS)^90^. This subscale of OSNANS consists of four questions with answers on a 5-point Likert scale (from 1, mostly inaccurate, to 5, mostly accurate). The items were translated into Polish and then back-translated into English by another translator. An examples items from the questionnaire are: “To degree to which I am able to supervise the work of other people”, “To degree to which I am able to organize and direct of activities of others“. This scale exhibits good psychometric properties^90^. The one-factor structure of this subscale was confirmed, explaining 73.85% of the variance of the satisfaction of the need for power construct. The Cronbach’s alpha reliability coefficient of this subscale in this research was .88.

#### Organizational pride

The Organizational Pride Scale ^91^ was applied to examine organizational pride. It encompasses seven items divided into two dimensions: emotional pride (four items) and attitudinal pride (three items). The answers are given on a 5-point Likert (from 1 = strongly disagree to 5 = strongly agree). The items were translated into Polish and then back-translated into English by another translator. The one-factor structure of this measure was supported, explaining 72.61% of the variance of this construct. An example item of this measure is: “I am proud to work for my company”. The good reliability of this tool was confirmed using Cronbach’s alpha, which was 0.94.

#### Dark triad traits

The Polish version of the Dirty Dozen scale^91^ was applied to measure dark triad personality traits. The three-factor structure of this tool—consisting of narcissism, Machiavellianism, and psychopathy—was confirmed. Each factor was assessed through four items that were answered on a 5-point Likert scale (from 1 = strongly disagree to 5 = strongly agree). This tool has satisfactory psychometric properties for the Polish population regarding internal consistency and test–retest reliability. Example questions about narcissism include: “I tend to want others to admire me”, psychopathy: “I tend to lack remorse”, and Machiavellianism: “I tend to manipulate others to get my way”. In this study, the reliability, measured by the alpha Cronbach’s coefficient, was 0.90 for narcissism and Machiavellianism and 0.79 for psychopathy.

## Results

### Statistical analysis and preliminary results

The sociodemographic data of the study sample are shown in Table 1.

Potential problems with multicollinearity were verified by a variance inflation factor. Achieved values less than the threshold (5; Table 2)^92^ was the premise for admitting the accepted level of multicollinearity as non-problematic. AMOS was used to conduct confirmatory factor analysis (CFA) to verify the potential for common method bias and to assess the discriminant validity of the research constructs. The Harman method of one non-rotated factor solution in CFA, which explained the 30,34% common variance, implied the absence of a problem with common method bias^93^. An additional argument for the lack of method bias was that the research model with a one-factor solution was poorly fitted to the data (see Table 3).

**Table 2:** Descriptive statistics (*N* = 1000).

Variable	Minimum	Maximum	Mean	Standard deviation	Skewness	Kurtosis	VIF
Narcissism	4	20	8.96	4.22	0.42	−0.85	2.32
Machiavellianism	4	20	7.78	4.00	0.79	−0.47	3.96
Psychopathy	4	20	8.22	3.63	0.62	−0.42	3.19
Opportunities to satisfy the need for power	4	20	16.05	4.17	0.03	− 0.83	1.2
Instrumental climate	4	20	10.77	4.20	− 0.21	0.21	1.14
Organizational pride	7	35	23.76	6.21	− 0.46	0.35	–

Variance inflation factor.

**Table 3:** The results of CFA for all measures used in the research measures (*N* = 1000).

Number of model	Factors	CMIN/DF	RMSEA	SRMR	GFI	CFI	TLI	NFI
One factor model	N, P, M, NP, IC, OP	16.77 (*p* = .000)	[0.126, 90% (0.123; 0.129)]	0.1704	0.68	0.74	0.70	0.73
Two factor model	N, P + M, NP, IC, OP	15.84 (*p* = .000)	[0.122, 90% (0.119; 0.125)]	0.1700	0.69	0.76	0.72	0.75
Three factor model	N + P + M, NP, IC, OP	18.99 (*p* = .000)	[0.134, 90% (0.131; 0.137)]	0.2326	0.65	0.71	0.66	0.70
Four factor model	N + P+M + NP, IC, OP	7.82 (*p* = .000)	[0.083, 90% (0.080; 0.086)]	0.1355	0.83	0.89	0.87	0.87
Five factor model	N + P+M + NP+ IC, OP	5.24 (*p* = .000)	[0.065, 90% (0.062; 0.068)]	0.0980	0.88	0.93	0.92	0.92
Six factor model	N + P+M + NP+ IC + OP	2.42 (*p* = .000)	[0.038, 90% (0.034; 0.42)]	0.0462	0.94	0.97	0.97	0.96

N,  Narcissism; P, psychopathy; M, Machiavellianism; NP, opportunities to satisfy the need for power; IC, instrumental ethical climate; OP, organizational pride.

Model fit was assessed using the following indicators: Chi-square/df (acceptable value < 5), RMSEA (acceptable value < 0.08), SRMR (acceptable value < 0.08), GFI, CFI, TLI, and NFI (acceptable values> 0.90).

The CFA confirmed that the six-factor solution fits the data well compared to alternative models (see Table 3), which provided the arguments to admit the research variables as separate constructs due to meeting the assumption about discriminant validity.

The R-Person correlation coefficients of the study variables are presented in Table 4. Narcissism, Machiavellianism, psychopathy, the opportunities to satisfy the need for power, and an instrumental ethical climate were positively intercorrelated. Psychopathy was weakly negatively related to organizational pride, narcissism was weakly positively correlated with organizational pride, and Machiavellianism was irrelevant for organizational pride.

**Table 4:** Values of r-pearson correlation coefficients between research variables (*N* = 1000).

	1	2	3	4	5	6	7	8	9	10	11
1. Narcissism											
2. Machiavellianism	0.74**										
3. Psychopathy	0.66**	0.82**									
4. Instrumental ethical climate	0.22**	0.24**	0.24**	.							
5. Opportunities to satisfy the need for power	0.32**	0.32**	0.29**	0.31**							
6. Organizational pride	0.07*	− 0.05	− 0.08*	0.03	0.29**						
7. Position held	− 0.15**	− 0.10*	− 0.04	− 0.03	− 0.36**	− 0.17**					
8. Seniority	− 0.06*	− 0.06	− 0.07*	0.05	− 0.02	0.01	− 0.01				
9. Seniority in present work	− 0.05	− 0.04	− 0.06*	0.04	0.08*	0.07*	− 0.09*	0.10**			
10. Educational level	0.02	− 0.01	− 0.04	− 0.09**	0.09**	0.04	− 0.20**	− 0.03	0.01		
11. Age	− 0.15**	− 0.13**	− 0.16**	− 0.01	0.01	0.09**	− 0.09**	0.25**	0.51**	− 0.08*	
12. Company size	0.01	− 0.05	− 0.07*	− 0.01	− 0.03	− 0.02	− 0.05	0.01	0.13**	0.07*	0.07*

*p < .05. **p < .01.

### Hypotheses testing

The hypotheses were tested using IBM SPSS (version 29) statistical software.

The study hypotheses were verified by applying model 58 from the Hayes PROCESS macro^94^, which refers to a moderated mediation model. The moderation effects were tested based on mean, dividing the sample into three groups: –SD, mean, and + 1 SD, reflecting “low,” “medium,” and “high” values on that variable^95^.

 Hypothesis 1 about the positive association between narcissism and organizational pride and the negative associations between Machiavellianism and psychopathy with organizational pride was supported. A positive, significant direct relationship between narcissism and organizational pride was observed (B = 0.30, t = 4.51, *p* = 001, CI [0.1677, 0.4259]), as was a negative direct relationship between Machiavellianism and organizational pride (B = − 0.26, t = − 2.89, *p* = 003, CI [− 0.4371, − 0.0839]) and the negative direct relationship between psychopathy and organizational pride (B = − 0.29, t = − 3.24, *p* = 001, CI [− 0.4481, − 0.1154]).

**Fig. 2:**
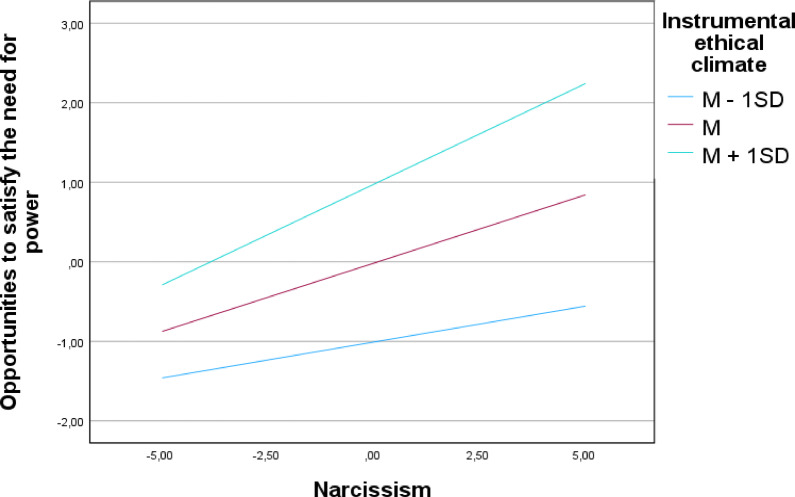
The moderating effect of employees’ perception of instrumental ethical climate on the relationship between narcissism and the opportunities to satisfy the need for power.

**Fig. 3:**
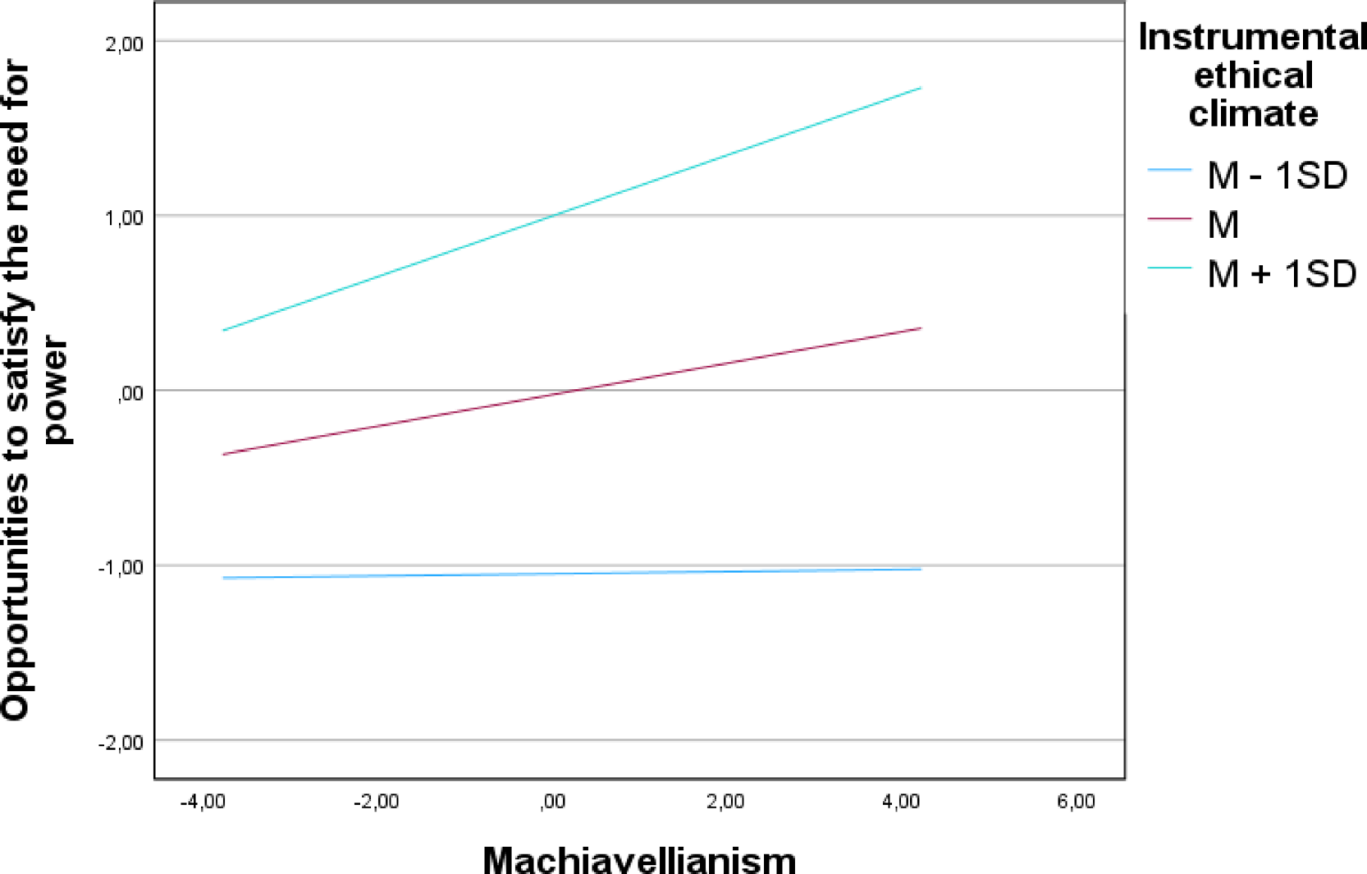
The moderating effect of employees’ perception of instrumental ethical climate on the relationship between Machiavellianism and the opportunities to satisfy the need for power.

**Fig. 4:**
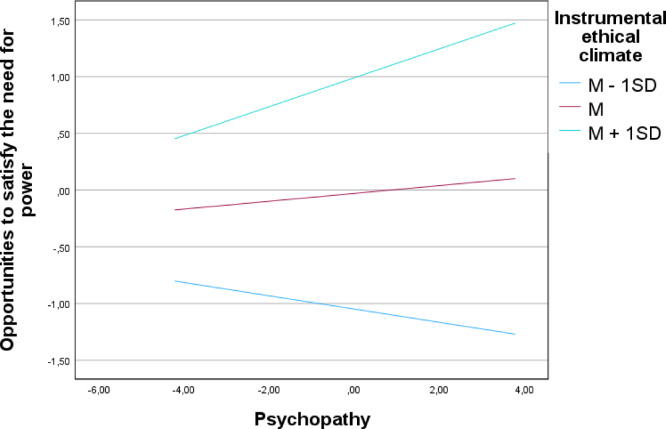
The moderating effect of employees’ perception of instrumental ethical climate on the relationship between psychopathy and the opportunities to satisfy the need for power.

Consistently with Hypothesis 2, an instrumental ethical climate moderated the relationships between dark triad traits and opportunities to satisfy the need for power. The results of the moderation analyses are shown in Table 5 and presented in Figs. 2 and 3, and 4. The moderating effect of narcissism and perception of instrumental ethical climate explained an additional 0.82% of the variance of opportunities to satisfy the need for power. In the same vein, the moderating effect of Machiavellianism and perception of instrumental ethical climate explained an additional 0.70% of the variance of opportunities to satisfy the need for power and the moderating effect of psychopathy and perception of instrumental ethical climate explained an additional 0.75% of the variance of opportunities to satisfy the need for power.

**Table 5:** Results of moderation analyses (*N* = 1000).

Hypotheses	Moderating variable	Interaction effect	Coefficient	t	*p*	LLCI	ULCI
H2 (opportunities to satisfy the need for power)	Instrumental climate	Narcissism x Instrumental climate	0.020	3.19	0.001	0.0079	0.0329
H2 (opportunities to satisfy the need for power)		Machiavellianism x Instrumental climate	0.021	2.93	0.003	0.0070	0.0349
H2 (opportunities to satisfy the need for power)		Psychopathy x Instrumental climate	0.022	2.94	0.033	.0074	0.0370
H3 (organizational pride)		Opportunities to satisfy the need for power x Instrumental climate	0.049	5.57	0.000	0.0323	0.0674

LLCI, 95% confidence interval (low); ULCI, 95% confidence interval (high).

All moderation effects are shown in Table 6. The relationships between narcissism and psychopathy and opportunities to satisfy the need for power were found in a group of employees with average and more than average perceptions of instrumental climate. The moderating effect of the perception of instrumental climate on the relationships between Machiavellianism and opportunities to satisfy the need for power, and between psychopathy and opportunities to satisfy the need for power, was observed only among employees with above-average perceptions of instrumental ethical climate.

**Table 6:** Moderating effects of dark triad traits on opportunities to satisfy the need for power and opportunities to satisfy the need for power on organizational pride for different values of instrumental ethical climate (*N* = 1000).

Instrumental climate	Effect	t	*p*	LLCI	ULCI
7 (− 1 *SD*)	0.079	1.32	0.185	− 0.0380	0.1958
11 (*M*)	0.161	3.15	0.017	0.0608	0.2609
15 (+ 1 *SD*)	0.243	4.29	0.000	0.1317	0.3538
Narcissism as independent variable and opportunities to satisfy the need for power as outcome variable					
7 (− 1 *SD*)	0.006	0.09	0.927	− 0.1260	0.1328
11 (*M*)	0.090	1.52	0.127	− 0.0258	0.2057
15 (+ 1 *SD*)	0.174	2.73	0.006	0.0492	0.2986
Machiavellianism as independent variable and opportunities to satisfy the need for poweras outcome variable					
7 (− 1 *SD*)	− 0.058	− 0.84	0.398	− 0.1944	0.0774
11 (*M*)	0.034	0.58	0.559	− 0.0814	0.1505
15 (+ 1 *SD*)	0.127	2.00	0.045	0.0027	0.2523
Psychopathy as independent variable and opportunities to satisfy the need for poweras outcome variable					
7 (− 1 *SD*)	0.273	4.42	0.001	0.1524	0.3747
11 (*M*)	0.473	9.76	0.000	0.3780	0.5682
15 (+ 1 *SD*)	0.673	11.46	0.000	0.5575	0.7878
Opportunities to satisfy the need for power as independent variable and organizational pride as outcome variable					

LLCI , 95% confidence interval (low); ULCI, 95% confidence interval (high).

**Fig. 5:**
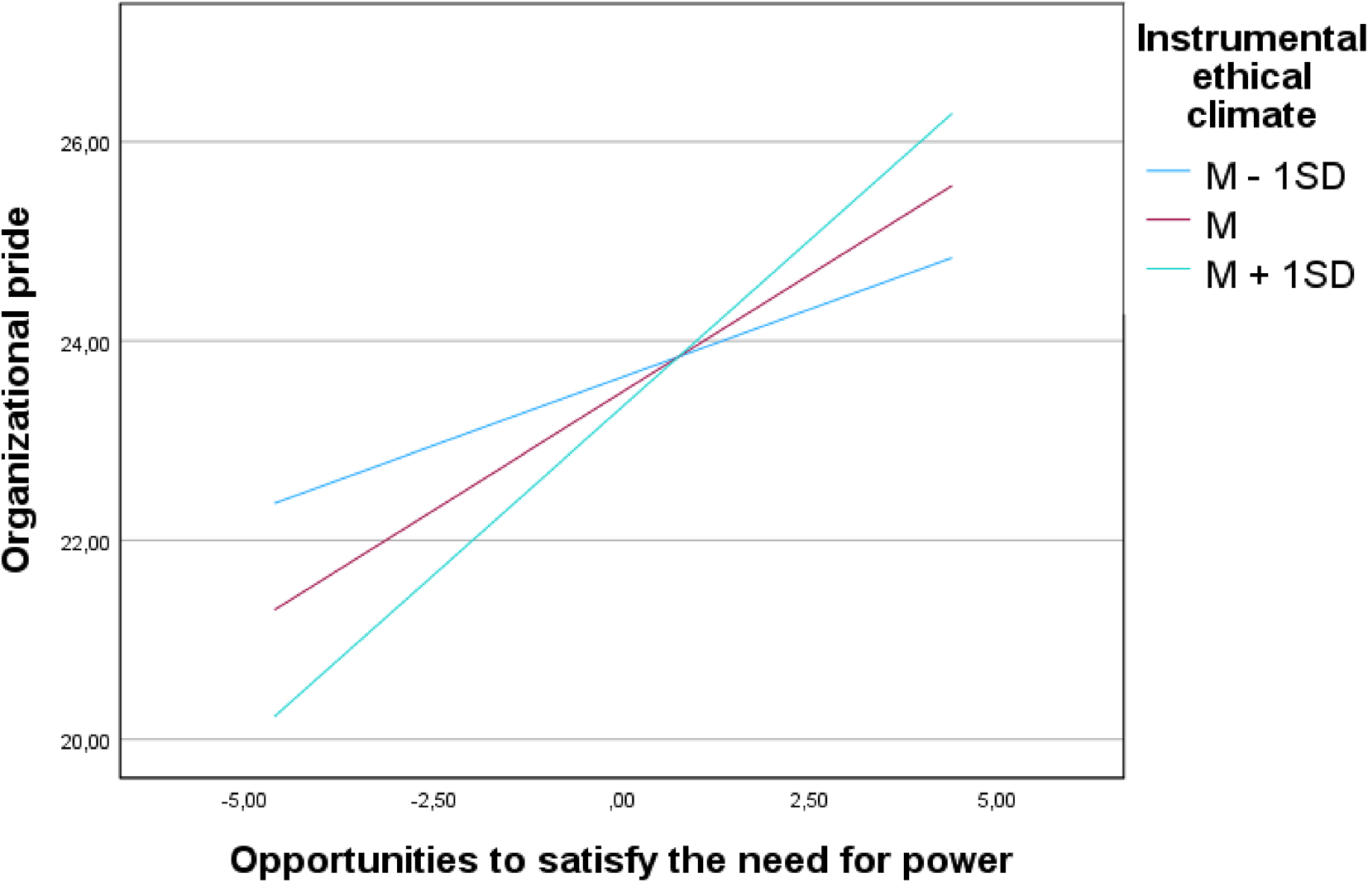
The moderating effect of employees’ perception of instrumental ethical climate on the relationship the opportunities to satisfy the need for power and organizational pride.

Hypothesis 3 about the moderating effect of instrumental ethical climate in the link between the opportunities to satisfy the need for power and organizational pride was supported (see Table 5), which is presented in Fig. 5. The moderating effect of opportunities to satisfy the need for power and perception of instrumental ethical climate explained an additional 2.60% of the variance of organizational pride.

Hypothesis 4 regarding the mediating role of opportunities to satisfy the need for power in the link between dark triad traits and organizational pride, moderated by an instrumental ethical climate, was partially confirmed. Conditional indirect effects for different levels of moderators in the relationship between dark triad traits on organizational pride through opportunities to satisfy the need for power as a mediator are presented in Table 7. Narcissism was indirectly related to organizational pride by opportunities to satisfy the need for power in employees with average and higher-than-average perceptions of instrumental climate. The indirect effect of Machiavellianism on organizational pride was statistically significant in a group of employees with a higher-than-average perception of instrumental climate. The indirect effect of psychopathy on organizational pride, through opportunities to satisfy the need for power, was statistically insignificant, regardless of the moderator’s level. None of the controlled variables was a statistically significant predictor of organizational pride. The model explained 16.51% (*R* = .406) of the variance in organizational pride.

**Table 7:** Conditional indirect effects of dark triad traits on organizational pride through the opportunities to satisfy the need for power as a mediator on the three levels of value of instrumental climate (*N* = 1000).

Instrumental climate	Effect	LLCI	ULCI	
7 (− 1 SD)	0.025	− 0.0062	0.0633	
11 (M)	0.081	0.0319	0.1329	
15 (+ 1 SD)	0.170	0.0869	0.2562	
Narcissism as independent variable and organizational pride as outcome variable				
7 (− 1 SD)	0.001	− 0.0436	0.0421	
11 (M)	0.042	− 0.0195	0.1061	
15 (+ 1 SD)	0.117	0.0197	0.2221	
Machiavellianism as independent variable and organizational pride as outcome variable				
7 (− 1 SD)	− 0.016	− 0.0608	0.0224	
11 (M)	0.016	− 0.0431	0.0777	
15 (+ 1 SD)	0.086	− 0.0051	0.1816	
Psychopathy as independent variable and organizational pride as outcome variable				

LLCI , 95% Confidence Interval (Low); ULCI, 95% Confidence Interval (High).

## Discussion

### Direct relationships between the dark triad and organizational pride

Following the P–O fit theory,^26^ this study aimed to examine the mechanisms behind the connection between Polish employees’ dark triad personality traits and their organizational pride. It was the first attempt to describe and explain the interaction of dark triad traits with the perception of instrumental ethical climate in explaining opportunities to satisfy the need for power as an antecedent of organizational pride. As was expected, the direct relationship between narcissism and organizational pride was positive in comparison to the negative direct relationship between Machiavellianism and organizational pride and between psychopathy and organizational pride. It corresponds with a previous study showing the propensity of employees with narcissistic traits to identify and attach to an organization^42,44^ and derive pride from it without the need to fill any additional criterion regarding appropriate ethical workplace and motivational factors. These differences probably lie in the specificity of the leading motivational aspects of individuals with narcissistic, Machiavellian, and psychopathic characteristics. Most of all, the major discrepancy between employees with narcissistic traits compared to Machiavellian and psychopathic ones regards affiliation needs^45^ and narcissistic admiration as an overarching motive of conduct^49^. It explains the propensity of workers with narcissistic traits to identify with the organization^48^, build attachment^42,44^, and feel pride as a potential strategy to protect from interpersonal rejection. Fulfilling the need to belong in narcissistic individuals requires a feeling of being part of something bigger and significant. Being a part of something meaningful and sharing the same values can protect them from interpersonal rejection, which they are afraid of and avoid. Additionally, it provides the premises for positive identification with the organization as a set of special workmates connected by similar values, needs, and desires, which differ from those of other organizations and may result in a feeling of being better than other organizations. It is a fruitful ground for workers with narcissistic characteristics to be admired, but on the communal, not individual, level, and additionally, for building a stronger group identity through negative reference to other companies as potential rivals^49^. It needs to be emphasized that the above-mentioned aspirations and needs of employees with narcissistic traits are still instrumental, not autotelic, and directed to self-enhancement, domination, and exploitation of others, but can serve as a tool to protect from social rejection.

Also, the positive relationship between narcissistic characteristics and organizational pride needs to be interpreted with more caution due to the potential presence of a social desirability effect, which is typical for individuals with narcissistic traits. As Kowalski et al.^96^ reported, socially desirable responding was positively correlated with narcissism and negatively correlated with both Machiavellianism and psychopathy. Additionally, it should be added that a positive moderate relationship between narcissism and organizational pride had a place in linear regression, but in pairwise correlation, this relationship was weak. It can be interpreted as a suppression effect due to the presence of controlled variables in the linear regression model. Another possible interpretation is partial collinearity, which occurs when a moderate or strong linear relationship exists between two or more predictor variables in a regression model. Narcissism was strongly positively correlated with Machiavellianism and on the borderline between moderate and strong correlation with psychopathy, which meet the criteria of partial collinearity. Machiavellianism and psychopathy were included as covariates in the linear regression model and could account for this effect. Partial collinearity can increase coefficient standard errors, making them less precise and potentially leading to non-significant p-values for important variables.

The negative correlation between psychopathy and organizational pride can be explained by their callousness and unemotional traits^97^, which makes the organization, in their eyes not worth attachment to and involvement with^43^. On the other hand, as shown, they can express pride in the organization, but only when they perceive the organization’s ethical standards as individual-oriented.

The negative effect of Machiavellianism on organizational pride can be caused by the lack of profit for workers with Machiavellian characteristics from this kind of attitude, for whom the most important thing is achieving selfish goals through manipulation, exploitation of others, and using Machiavellian tactics, and the workplace environment is a rather chance to achieve superiority over others than build organizational pride^31^.

### Dark triad and opportunities to satisfy the need for power - moderating role of instrumental climate

As indicated in previous research, in employees with dark triad traits, the satisfaction of the need for power was a crucial^60–62^ and conditional factor for the feeling of organizational pride. According to trait activation theory^66^, the stronger the instrumental climate was perceived by employees with dark personality traits, the bigger the probability of opportunities to satisfy the need for power was, which shows their propensity to interpret the ethical cues that came from the organization as an incentive to desire for power and stay in congruency with their drives and self-oriented approach.^14^. What deserves attention, despite the sensitivity of workers with dark triad traits on the influence of instrumental climate on their relationship with opportunities to satisfy the need for power, the change in perception of this climate in plus was more beneficial to workers with narcissistic than with Machiavellian and psychopathic traits. This discrepancy shows that employees with Machiavellian and psychopathic characteristics are less prone to search for ethical arguments in an instrumental climate to justify their striving for power satisfaction. Among Machiavellian employees, the tendency to manipulate and exploit others to gain power is the inherent element of their personality structure^31^. This conclusion stays in agreement with Spurk and Hirschi’s^11^ observations that employees with narcissistic and psychopathic traits use strategies to select a work environment in agreement with their preferences to compete^11^. In turn, workers with Machiavellian characteristics are probably convinced that, due to the manipulation strategy^12^, regardless of the ethical climate can achieve their purpose and manifest their superiority over others. In reference to workers with psychopathic traits, their low sensitivity to ethical organizational standards, despite coherence with their needs and values, may be the effect of their limitations of social learning resulting from their immorality, characterized by a profound lack of conscience and disregard for social norms. The moderation effects explain only a small proportion of the variance in organizational pride, suggesting caution in interpreting these results and in drawing theoretical and practical implications.

### Dark triad and organizational pride – indirect effects

Achieved results showed that the relationships between employees with narcissistic, Machiavellian, and psychopathic traits and organizational pride, besides the direct effects, are more complicated and can also have an indirect character. In the group of workers with narcissistic and Machiavellian traits in comparison to psychopathic ones, the perception of instrumental climate amplifies organizational pride by increasing the opportunities to satisfy the need for power. This means that the perception of instrumental climate may serve a beneficial function for organizational pride, by providing opportunities to satisfy the need for power, and this effect is stronger among narcissistic employees compared to Machiavellian ones, but did not occur among those with psychopathic characteristics.

It is essential to stress that instrumental ethical standards are well-suited for employees with dark triad traits; however, manifestations of these traits can be detrimental to other employees and the organization. Earlier studies have confirmed that, among Polish employees, the perception of instrumental climate is negatively correlated with affective commitment, person-organization fit, meaning in work^98^, but positively correlated with job stress and organizational cynicism^30^.

P-O fit theory offers a good theoretical framework^26^ to explain this study’s findings, emphasizing the positive effect of congruence between the values and needs of dark triad employees and the ethical preferences and demands on their attitudes in the workplace, which ultimately bears fruit in organizational pride. In the line of trait activation theory^52^, environmental cues flowing from the perception of an instrumental ethical climate as coherent with the ethical code of conduct and selfish orientation^14^ of employees with dark triad traits amplify the positive effect of their perception of opportunities to satisfy the need for power and the positive effect of the ability to fill this need on organizational pride. The obtained results show how contextual ethical environment factors can stimulate the expression of certain traits in the workplace and how their manifestations can yield beneficial outcomes despite these traits being generally antisocial and unwanted^31^.

### Theoretical contribution

The dark personality traits potent in shaping pride in the organization were tested for the first time in an ethical organizational environment and in the presence of a motivational factor (opportunities to satisfy the need for power) necessary for this kind of attitude toward the organization. What requires emphasizing not only direct mechanisms but also more complex ones underlying the link between dark triad traits and organizational pride was confirmed.

This study contributes to earlier findings^11^ through the recognition that an appropriate ethical environment in interaction with dark triad personality traits suited to this context can be beneficial both for employees and the organization, despite antisocial and amoral behaviors associated with expressing these traits. Moreover, it was confirmed that this kind of antisocial attitude in an instrumental climate can be tolerated. Recent studies showed that a competitive climate is wanted and expected for this group of employees^11^, and this study was the next step, showing that matching workers with dark triad traits, an instrumental climate can give them a chance to satisfy the need for power and build pride in the organization.

The relatively weak effect of moderating the perception of instrumental ethical climate on the relationship between each dark triad trait and the need for power suggests that these results should be interpreted with caution. It is worth noting the differences in susceptibility to the perception of instrumental climate across the dark triad in the context of opportunities to fill the need for power. The strongest effect was observed in employees with narcissistic traits, but among workers with Machiavellian and psychopathic inclinations, this effect was weaker. It may be interpreted in terms of the specificity of manifestations of three different dark triad traits in the workplace. As this study demonstrated, employees with Machiavellian traits, likely due to their beliefs in their manipulation skills, cunningness, and adaptability, are less susceptible to incentives stemming from their perception of instrumental ethical standards to satisfy the need for power. Also, psychopathic workers, because of their amorality, have limited susceptibility to react according to the requirements of an ethical organizational environment. On the other hand, the Dirty Dozen Scale’s low discriminant validity could suppress potential differences in the strength of the moderating effects, as well as differences in the associations between every dark triad trait manifestation and organizational pride.

Consistent with earlier studies, reported narcissism, as related to positive outcomes in the workplace; it was found that the narcissism of employees is directly positively related to organizational pride. Additionally, in line with research outcomes reporting negative associations between Machiavellianism and affective commitment and psychopathy and affective commitment in this study, I found analogous results using organizational pride instead of affective commitment. It is possible that using a measure other than the Dirty Dozen Scale, which is characterized by better discriminant validity to verify, especially Machiavellianism and psychopathy, would strongly expose the differences in the relationships between particular manifestations of dark triad traits and organizational pride.

It shows that in this group of employees, experiencing organizational pride is possible under the condition of instrumental ethical standards in the organization and their opportunities to satisfy the need for power.

### Practical implications

The practical implications of the findings include recommendations on ethical standards that serve as a foundation for fostering organizational pride among employees with dark triad traits and for addressing the potential threats they pose. On the one hand, organizations may provide opportunities to satisfy the need for power through the possibility of promotion and by fostering an ethical climate that meets the needs of individuals, not the collective, and by emphasizing the realization of self-interests, not altruistic goals. On the other hand, it is essential to emphasize that this would be detrimental to the well-being of other employees, making it difficult to promote prosocial behaviors and potentially decreasing cooperativeness, while amplifying organizational cynicism^98^. In companies, it can create a dilemma between the individual’s and the community’s interests, and the need to find a healthy balance between the two. This means that representatives of organizations and human resource specialists can create conditions that enable them to meet their needs without harming coworkers or the organization.

A process for promotion should be standardized and established, then communicated to employees for transparency, so everyone knows the rules and requirements for promotion. Within the promotion process, aspirations and ambitions connected with the need for the power of employees with the dark triad traits should be recognized, and in case of failure, organizations should create the opportunity to dominate, control, and influence in other ways, such as, for example, in a project manager function or additional duties fitted to these traits. A recruitment strategy that includes personality evaluation and methods to verify the alignment between the organization’s needs and values and those of recruited individuals can be implemented. This would help ensure that an appropriate employee fits with the organization, increase the likelihood of bonding with and being proud of the organization, and limit turnover intentions. Organizations characterized by a caring ethical climate^13^ focused on altruistic values such as support, affiliation needs, satisfying the needs of every employee, empathy, and cooperation should avoid hiring candidates with dark triad traits due to incongruency in fitness between the parties. They probably should instead search for candidates with opposite personality profiles, where humility, agreeableness, and a desire to fulfil affiliation needs are advantages, ensuring mutual fitness.

### Limitations and future research

The main limitation of this study is its cross-sectional, non-longitudinal design, which prevents presenting results from a cause-and-effect perspective. Future investigations should have a longitudinal character and consider the caring, ethical climate, which is opposite to the instrumental climate^13^, and the predispositions and preferences of employees with dark triad traits. It could show whether and how narcissistic, Machiavellian, and psychopathic workers try to adapt to ethical requirements contrary to their ethical code of conduct, where, instead of power, rivalry, selfishness, exploitation of others, or manipulation, subordination, cooperation, altruism, and helping others are expected.

A multi-source design encompassing self-report and additional superior evaluations should be conducted to identify potential consistency or discrepancies in results from both sources, thereby avoiding the potential social desirability effect detected in individuals with narcissistic traits^96^.

Other personality conceptions, like Big Five or HEXACO, and the need for achievement and affiliation, besides dark triad traits and the need for power, should be tested to show a more holistic picture of interactions and potential coherence between personality, motivation, and ethical climate in explaining organizational pride. Also, two types of narcissism, such as vulnerable (covert) and grandiose (overt), as two different manifestations of this trait, should be taken into consideration to verify a potential connection with organizational pride. Previous studies showed different relationships between vulnerable and grandiose narcissism and workplace outcomes. For example, Dåderman and Kajonius^99^ found that vulnerable narcissism is positively related to counterproductive work behavior and negatively correlated with task performance, but grandiose narcissism was unrelated to counterproductive work behavior and negatively correlated with task performance. On the other side, the Dirty Dozen Scale, used for the verification of narcissism in this study, well captured both types of narcissism^100^, which suggests potential similarity in the relationships between vulnerable and grandiose narcissism and organizational pride. This measure exhibits good psychometric properties but has some weaknesses that could affect research results. First of all, the “dark core” often explains a significant portion of the variance, leaving less unique variance for individual traits, which can complicate the interpretation of subscale scores. Additionally consists of common elements more characteristic of Machiavellian and psychopathy than narcissism^15^. At last, encompass a too narrow and insufficient coverage of certain traits essential to psychopathy^101^.

Finally, the other sociocultural context should be considered as a factor that may determine the relationships between dark triad traits and different indicators of attitude toward the organization. Poles are a religiously inclined nation that represents a high degree of religious homogeneity, because the vast majority are representatives of the Roman Catholic Church^102^. This is reflected in the nearly strong positive correlation between the law and code ethical climate and the rules ethical climate^30^, which may indicate how religious ethical standards in Poland can influence the ethical rules and values within companies. This implies that future research should be conducted on this topic in more diverse sociocultural contexts, encompassing a wider range of religious diversity and a broader spectrum of secular backgrounds. It seems essential to consider the cultural context, based on Hofstede’s typology^103^, as a potential factor that may influence the perception of the ethical climate. It seems possible that in more individualistic countries, the propensity to perceive the ethical climate as instrumental may be stronger than in collectivistic ones. From this point of view, Poland’s cultural profile indicates moderate individualism^104^, suggesting a potential lack or weak influence of cultural factors on the perception of ethical climate as instrumental. Among Polish employees, a positive, moderate relationship was found between independence ethical climate and caring ethical climate, and a negative, weak relationship between independence ethical climate and instrumental ethical climate, suggesting a rather inverse tendency^30^.

## Data Availability

All data is available for the individual request directed to the author at the correspondence address: marwnu@amu.edu.pl.
